# Estrogen is not neuroprotective in a rodent model of optic nerve stroke

**Published:** 2007-10-09

**Authors:** Steven L. Bernstein, Zara Mehrabyan, Yan Guo, Nima Moianie

**Affiliations:** 1 Department of Ophthalmology,; 2 Anatomy and Neurobiology,; 3 Genetics, University of Maryland School of Medicine, Baltimore, MD,; 4 Department of Anesthesiology, University of Maryland School of Medicine

## Abstract

**Purpose:**

Nonarteritic anterior ischemic optic neuropathy (NAION) is an optic nerve (ON) infarct of retinal ganglion cell (RGC) axons, and the most common cause of ON-related sudden vision loss. Estrogen has been previously proposed as a neuroprotective treatment for central nervous system ischemia. We evaluated estrogen's potential in post-ON infarct treatment to reduce neuronal loss following a model of NAION, rodent anterior ischemic optic neuropathy (rAION).

**Methods:**

We used the rat rAION model, coupled to array and northern analyses, to evaluate estrogen-associated, early post-infarct retinal gene expression changes. rAION was induced in ovariectomized female rats, which were then treated with either estrogen or vehicle. Stereological analysis of post-rAION RGC numbers was performed, using retrograde RGC fill-labeling with fluorogold.

**Results:**

rAION induces an early increase in estrogen expressed transcript-1 (EET-1), but EET-1 expression is not affected by systemic estrogen pretreatment. Post-rAION, there is no significant increase in RGC numbers in estrogen treated animals compared with vehicle-treated controls. Estrogen treatment following stroke does not increase preservation of ON structure, compared with vehicle controls.

**Conclusions:**

While the rAION-axonal stroke model is a useful adjunct for evaluating potential AION neuroprotective treatments, post-stroke estrogen administration does not appear neuroprotective in this form of central nervous system insult. Similarly, estrogen is likely to be ineffective in improving ON structural integrity following an ischemic infarct.

## Introduction

The optic nerve (ON) is a central nervous system (CNS) tract comprised of the axons of retinal ganglion cells (RGCs), and myelinated by oligodendrocytes. Nonarteritic anterior ischemic optic neuropathy (NAION), an infarct of the ON, is produced by sudden RGC axonal ischemia [1]. NAION is the major cause of sudden ON-related vision loss in individuals over 50 years old [2]. NAION clinically resembles other CNS white matter strokes [3], with isolated dysfunction and physiologically demonstrable deficits [4]. NAION produces RGC loss and ON gliosis, with sparing of other retinal neurons [3,5]. No currently effective measures exist to prevent or treat this condition [6].

We recently reported on a new rodent NAION model (rAION) [7], which utilizes laser photoactivation of rose Bengal to generate superoxide radicals [8]. These radicals selectively damage ON capillary vascular endothelium, producing predictable and reproducible levels of isolated ON ischemia. rAION resembles clinical NAION by many parameters [7,9]. Similar to other CNS strokes, isolated ON stroke produces a biphasic gene response, with rapid (<1 day) and reactive (>3 days) tissue changes [7,10].

Estrogen is a sex steroid with neuroprotective effects in a variety of neural insults [11-13]. Women of pre-menopausal age have a significantly lower incidence of stroke than either men or post-menopausal women [14,15]; post-menopausal women have a poorer post-stroke recovery than men [16], and female animals subjected to ischemia have less overall CNS damage than males [17], suggesting that circulating estrogen may be neuroprotective against stroke. Estrogen administration can modify ischemia-related proteins in neuronal cell culture [18], and has been shown to be effective in reducing the area of stroke-related damage in a model of global CNS ischemia [19]. Estrogen administration can also protect RGCs following global retinal ischemia [20]. We have identified several genes whose expression is known to be affected by estrogen administration [21]. We were able to do this by using a microarray-based analysis of retina-expressed genes at early times (1–3 days) post-rAION in male rats by Affymetrix array analysis, and confirming expression by both real-time quantitative polymerase chain reaction and northern analysis (Bernstein et al., manuscript in preparation). Additionally, we initially evaluated the effects of estrogen pre-infarct administration using a “sighting study” on a small number (n=3) of animals by serial step-cut sections. These limited data suggested that estrogen could be a potentially useful neuroprotective agent following RGC axonal ischemia. However, effective post-stroke neuroprotection remains a problem that must ultimately be resolved by in-vivo analysis. Thus, we directly evaluated the neuroprotective effects of estrogen post-treatment after axonal stroke, using a statistically valid number of animals and modern stereological analysis [22].

## Methods

### Animals

All animal procedures were approved by the University of Maryland Baltimore institutional animal care and utilization committee before experimentation. Female ovariectomized Sprague-Dawley albino rats (105–120 g) were obtained from Charles River animal facility (Charles River, MA). Animals were kept in the University of Maryland Baltimore animal facility and were given food and water ad libitum.

### rAION induction

Prior to induction, animals were anesthetized using a mixture of ketamine/xylazine (80 mg/4 mg/kg). A fundus contact lens was placed to visualize the normal rat retina and ON. rAION was induced in anesthetized animals using tail vein intravenous injection of RB (2.5 mM). This was followed by ON laser illumination with a frequency doubled YAG laser (535 nm; Iridex Corporation; Mountain View, CA), coupled to a slit-lamp (Haag-Streit; Koeniz, Switzerland). Laser beam diameter was 500 μm/12 s. Previous histological studies have shown that this treatment level produces an ON lesion, resulting in an isolated 60%–75% RGC loss (data not shown). Pre- and post-induction, all eyes were photographed through a Haag-Streit slit lamp using an eyepiece adaptor (Edmund Scientific, Barrington, NJ) coupled to a Nikon D1X at 2.4 mPx, with automatic light correction, and 800 ASA.

### Post-optic nerve stroke estrogen treatment

rAION was induced in ovariectomized female rats (120–150 g; 75 days average age at induction). Induction levels were adjusted to produce a predicted loss of 60%–75% RGC loss (data not shown). Immediately after induction, animals were injected with either 50 μg/kg 17-estradiol (Wyeth/Ayerst; Phila, PA) in sesame oil (n=8 animals) or sesame oil vehicle (n=9 animals). An initial 'priming' dose of 5 µg/kg was given one day before induction in estrogen treated animals (G. Hoffman, personal communication).

### Retinal ganglion cell-retrograde labeling

Two weeks post-induction, animals were anesthetized with 10% chloral hydrate (3.5 ml/kg), and the skull skin infiltrated with 1% lidocaine. After skull exposure, 2 μl of 2% fluorogold (Molecular Probes, Invitrogen; Carlsbad, CA) in 0.9% saline was stereotactically injected into each side of the pretectum, using a stereotactic frame with digital readout (Stoelting Corp; Wood Dale, IL). Two weeks post-injection (28 days post-induction), animals were euthanized using deep pentobarbital anesthesia, and perfused with 4% paraformaldehyde-phosphate buffered saline (PF-PBS). Eyes werer enucleated, and the ON and retinae post-fixed 24 h in 4% PF-PBS. ONs were post-fixed in paraformaldehyde-glutaraldehyde.

### Retinal ganglion cell stereology

Post-fixed retinas were flat mounted using fluorescent mounting medium. Retrograde fluorogold-labeled RGCs were identified using a Nikon Eclipse E800 compound microscope with a 410 nm excitation filter/450 nm pass filter cube. We used a 20X air objective, of low numerical aperture, to give a depth of field sufficient to penetrate the nerve fiber and RGC layers. RGCs were counted using a computer-driven microscope stage that was, controlled by an optical fractionator linked to a stereological imaging package (Stereoinvestigator; Ver.6.0; Microbright-field, Bioscience, Williston, VT). Stereological analysis was performed using the Neuroleucida 6.0 program (Microbright-field), using a sufficient number of random sites within each defined region. At least 800 cells, at a minimum of nine sites, were counted per retina. This is greater than the number required by the Schmitz-Hof equation for statistical validity [22].

### Optic nerve histology

Fixed ON tissue was impregnated with uranyl acetate, stained with toluidine blue, and embedded in Epon. One-half micron sections were cut and stained with toluidine blue. ON sections were photographed using a Nikon D1X at 2.4 mPx.

## Results

**Figure 1:**
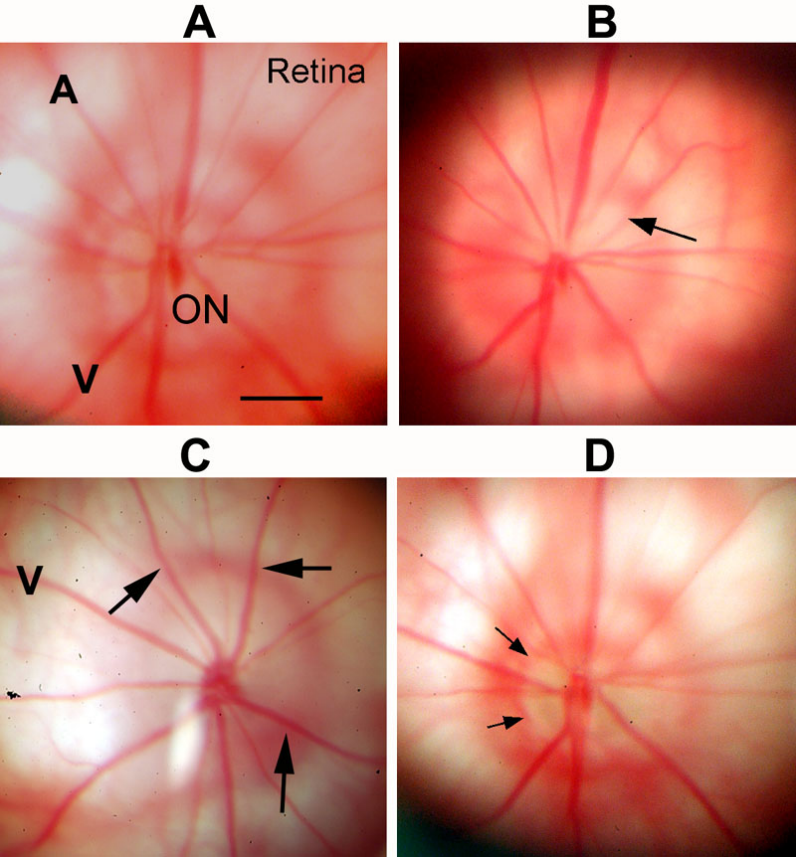
Appearance of estrogen- and vehicle treated retina and optic nerve before and after induction of rodent anterior ischemic optic neuropathy

**Figure 2:**
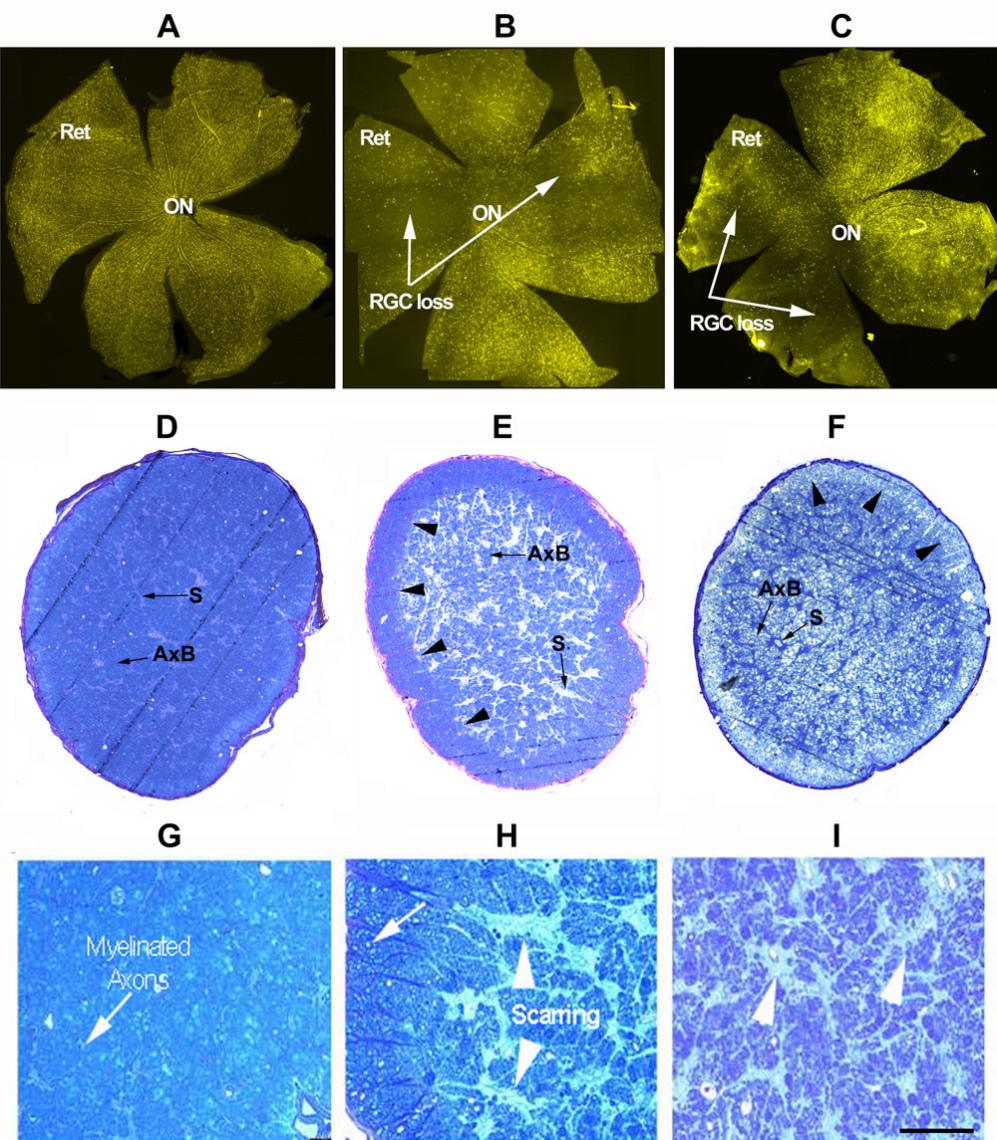
Histological comparison of retinal ganglion cells and optic nerves from naive, estrogen-treated, and vehicle treated animals following rodent anterior ischemic optic neuropathy induction

Estrogen does not change the appearance of retina post-rAION. The intra-ocular portion of the ON (Figure 1A) in a naive rodent eye is flat against the retina (Figure 1A), with distinct borders. Rodent AION typically produces ON edema 1 day post induction, followed by edema resolution and eventual ON atrophy [7]. The intraocular portion of the ON 2 days post-rAION induction in estrogen-treated animals (Figure 1B; same eye as in Figure 1A) and vehicle-treated (Figure 1C) animals showed IN edema. This was marked by both blurring of optic disk margins (arrow, Figure 1B), and elevation of the radial retinal vessels emerging from the ON (arrows; Figure 1C). There were no significant differences in retinal appearance between estrogen- and vehicle-treated induced eyes (n=17 eyes). There were no gross apparent changes in the ONs of contralateral (naive) eyes (data not shown). ON atrophy was detectable by post-induction day 21 (Figure 2C), typified by ON atrophy and pallor in all rAION-induced eyes, regardless of treatment (Figure 1D).

**A**: Pre-induction. The retina (Retina), optic nerve (ON) and retinal vessels (A and V) are normal in appearance. **B**: Same eye as **A** two days after-rodent anterior ischemic optic neuropathy (rAION) and estrogen treatment. The ON disk margin is blurred and edematous (arrow). **C**: rAION-induced+, vehicle-treated eye, two days post-induction. The ON is edematous, demonstrable as an alteration in direction of the retinal vessels emerging from the ON (arrows). **D**: ON appearance in estrogen treated animal 21 days post-rAION induction. The ON disk margin is apparently shrunken, with marked pallor (arrows). The scale bar represents 250 μm.

**A**, **D**, **G** are naíve animals, **B**, **E**, **H** are estrogen-treated animals, and **C**, **F**, **I** are vehicle treated animals. **A**-**C**: Distribution of fluorogold labeled retinal ganglion cells (RGC). Retinas from naive controls (**A**) have fluorogold labeled RGCs distributed over the retinal surface, with increased RGC numbers near the optic nerve (ON). At 28 days after rodent anterior ischemic optic neuropathy (rAION) induction, there is a regional loss of labeled RGCs in the estrogen-treated retina (**B**, arrows), with preservation of RGC distribution in the inferior wing of the retina. A similar regional RGC loss is seen in the retina of vehicle treated animals (**C**: arrows). **D**-**F**: Low power magnification of toluidine stained ON cross-sections (0.5 μm). **D**: Naive control. Axonal bundles (AxB) are evenly distributed over the surface, with thin axonal septae (s). **E**-**F**: ONs from rAION induced estrogen-treated (**E**) and vehicle treated (**F**) animals. There is a marked central loss of axons with increased septal thickness, and shrinkage of the axonal bundles (AxB). The majority of intact axons are present peripherally, with the greatest loss centrally. **G**-**I**: High power magnification of toluidine stained ON cross-sections (0.5 μm) from naive control (**G**), estrogen-treated animal (**H**), and vehicle-treated control (**I**) ONs show tightly packed myelinated axons (arrow) with thin septae. ONs from estrogen and vehicle-treated rAION induced animals show axonal loss and ON central scarring, demonstrable as increased septal thickening (arrowheads), with intact peripheral axons (arrow) and reduction in axonal bundle size. The scale bar represents 50 μm.

### Post-induction estrogen is not neuroprotective in axonal stroke

Fluorogold filled RGCs in non-induced control (naive) animals were distributed across the retinal surface (Figure 2A, ret). Relatively increased RGC numbers were seen centrally, closer to the ON (Figure 2A). Post-rAION induction, a regional loss of fluorogold labeled RGCs were seen in both estrogen treated (Figure 2B, arrows), and vehicle-treated (Figure 2C) retinas. There was relative sparing of RGCs in individual regions (compare inferior quadrant in Figure 2B and superior quadrant in Figure 2C with quadrants in naive control; Figure 2A). The loss of fluorogold labeled RGCs were similar in both vehicle- and estrogen-treated animals (compare Figure 2B and Figure 2C).

RGC axon loss patterns were similar in both rAION-induced vehicle and estrogen-treated animals. This was apparent in low magnification cross-section (Figure 2E,F) compared with naive control eyes (Figure 2D). Axonal loss was marked centrally in ONs from both estrogen and vehicle treated animals, with preservation greatest peripherally (Figure 2E-F; arrowheads). A higher power magnification of the same sections revealed a loss of individual axons, (compare Figure 2G with Figure 2H-I), and central scarring. There were regions of intact, myelinated axons, particularly in the ON periphery (Figure 2H; arrow). Overall loss of ON axons was similar in both vehicle controls and estrogen treated animals (compare Figure 2G with Figure 2H,I). These results suggest that post-stroke estrogen does not preserve RGCs in a regional fashion, nor does it provide ON protection against rAION-induced axonal loss.

### Stereological analysis of associated retinal ganglion cell loss in estrogen and vehicle controls after induction of rodent anterior ischemic optic neuropathy

**Figure 3:**
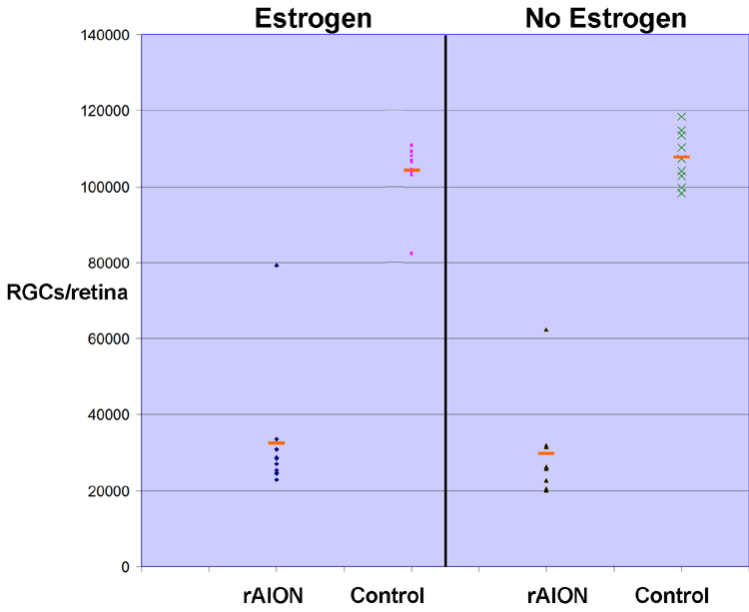
Stereological analysis after rodent anterior ischemic optic neuropathy induction retinal ganglion cell loss in estrogen- and vehicle-treated animals

Stereological analysis revealed an average of 102,500–108,000 RGCs in control (uninduced) retinas for both estrogen and vehicle groups (Figure 3, compare control numbers for vehicle-treated and estrogen-treated animals). Following rAION, a 25%–75% RGC loss was demonstrable in estrogen treated animals post-infarct as; compared with contralateral control (naive) eyes (Figure 3, estrogen). A similar RGC loss was seen in vehicle-treated animals as, compared with contralateral naive control eyes (Figure 3, compare rAION and control results in the vehicle group). There was an average 65% loss of RGCs in estrogen-treated animals and 70% RGC loss in vehicle treated animals. This difference is not statistically significant (p<0.15; Wilcoxon rank sum test).

Immediately after rodent anterior ischemic optic neuropathy (rAION) induction, animals received an intramuscular injection of either 50 μg/kg 17-estradiol, or vehicle (sesame oil) as described in Methods. Retinal ganglion cells (RGCs) were retrograde-labeled with fluorogold, and animals euthanized 28 days post-induction. Retinas were post-fixed, flat mounted and analyzed as described in Methods. A 65% average RGC loss is seen in estrogen treated animals, compared with a 70% RGC loss in vehicle treated animals. Control eyes: contralateral (naíve) eyes from each treatment group. Red bars indicate the mean RGC count for each group.

## Discussion

Following rodent ON stroke, both early (1 day) and later retinal gene expression changes occur [7], suggesting that isolated axonal ischemia causes a variety of changes in the RGC neuron and its surroundings. We hypothesized that genes whose relative expression alter after estrogen administration, and which also rapidly change their activity following ON infarct, could implicate estrogen-associated neuroprotective processes in the retina. The retina is an estrogen target [23]. Genes sensitive to estrogen administration are likely to be crucial in the early post-stroke period. Genes fulfilling these criteria include three RGC-expressed genes, HSC70, HSP90 α, and Brn3b [24-26], which we previously demonstrated rapidly change following rAION induction [7].

We also previously identified a rapid post-rAION increase in the retinal expression of estrogen-enhanced transcript-1 (EET-1; data not shown). EET-1 plays an important role in increasing levels of tumor necrosis factor α and other inflammatory cytokines [21], suggesting that estrogen could regulate post-rAION associated inflammation, by modulating tumor necrosis factor-alpha associated glial and microglial inflammatory processes that are important in reducing post-stroke morbidity [27,28]. However, we also determined that retinal EET-1 expression is not estrogen responsive (data not shown), suggesting that in the CNS and retina, estrogen does not modulate the inflammatory roles of EET-1 following infarct. These data, coupled with previous reports of estrogen-associated neuroprotection following retinal ischemia [20], and our earlier sighting study, supported our rationale for evaluating estrogen's potential as a neuroprotectant after axonal infarct.

It is possible that sex-related differential gene expression could result in sex associated differences in estrogen-related neuroprotection. However, estrogen receptors and EET-1 are both expressed at significant levels in male retinas [29]. EET-1 is also not estrogen-responsive in female CNS [30], which suggests that the current findings are not likely to be sex-dichotomous.

This study used ovariectomized animals, with estrogen administered post-stroke. Numerous trials have demonstrated that estrogen can have neuroprotective effects when given for a sufficiently long time before CNS insult, but a prior treatment approach is unlikely to be useful for clinical stroke therapeutics, which typically utilize therapy after the insult. Additionally, recent studies have not demonstrated that exogenous estrogen administration improves incidence or morbidity in stroke [31]. Thus, it is unlikely that estrogen is significantly neuroprotective when given after CNS axonal infarction in either male or female animals.
